# Frequency and Clinical Characteristics of Unselected Korean Gastric Cancer Patients with a Germline *CDH1* V832M Mutation

**DOI:** 10.7150/jca.36513

**Published:** 2020-01-01

**Authors:** Saeam Shin, Yoonjung Kim, Jin Kyung Lee, Kyung-A Lee

**Affiliations:** 1 Department of Laboratory Medicine, Yonsei University College of Medicine, Seoul, Korea;; 2 Department of Laboratory Medicine, Korea Cancer Center Hospital, Korea Institute of Radiological and Medical Sciences, Seoul, Korea;; 3 KIRAMS Radiation Biobank, Korea Institute of Radiological and Medical Sciences, Seoul, Korea.

**Keywords:** * CDH1*, E-cadherin, gastric cancer, hereditary gastric cancer, germline mutation

## Abstract

**Background:** Germline mutations in *CDH1* are associated with hereditary and early onset- diffuse gastric cancer. However, the frequency of *CDH1* germline mutation in unselected gastric cancer cases is not well established.

**Aim:** The aim of this study was to investigate the frequency and clinical characteristics of germline *CDH1* V832M mutation carriers in unselected Korean gastric cancer cases.

**Methods:** Direct sequencing was performed to determine the presence of *CDH1* V832M in 305 unselected Korean gastric cancer patients. Lauren's histologic type, family history of gastric cancer, and age of cancer diagnosis were compared between V832M carriers and non-carriers.

**Results:** In the study population, seven gastric cancer patients (7/305, 2.29%) were found to have the *CDH1* V832M mutation. The *CDH1* V832M mutation carrier state was not significantly associated with phenotypes including Lauren's histologic type, family history of gastric cancer, age of cancer diagnosis, and other cancer history in a patient.

**Conclusion:** This study demonstrates that the germline *CDH1* V832M mutation is common in sporadic, late onset, and intestinal type gastric cancer as well as familial, early onset, and diffuse type gastric cancer. Our finding suggests that guidelines for managing *CDH1* mutation carriers should be refined through additional data on penetration according to *CDH1* mutation type in sporadic cases.

## Introduction

Hereditary diffuse gastric cancer (HDGC, OMIM #137215) is an autosomal dominant cancer predisposition syndrome. Germline mutation in the E-cadherin gene (*CDH1*) is associated with familial cases of diffuse type gastric cancer and lobular breast cancer 1. Patients with HDGC characteristically develop diffuse gastric cancer at a young age; the average age for gastric cancer diagnosis is 37 years 2. The cumulative risk of gastric cancer for pathogenic *CDH1* mutation carriers by age 80 years is estimated to be 70% (95% CI, 59% to 80%) for males and 56% (95% CI, 44% to 69%) for females 1. Because of the high mortality from invasive cancer, early prophylactic total gastrectomy is recommended for *CDH1* truncating mutation carriers in current clinical guidelines 2,3. However, information about penetration and cancer risk of missense mutations, which accounts for 30% of total mutations found in HDGC families, is still insufficient 4. Moreover, as panel testing using next-generation sequencing increases in popularity, the likelihood of detecting *CDH1* germline mutations in patients not meeting HDGC diagnostic criteria is increasing and genetic counseling for these patients with missense mutations would confer a significant clinical burden 5,6.

Recently, we identified a *CDH1* V832M [NM_004360.4:c.2494G>A, NP_004351.1:p.(V832M)] missense variant in two out of 107 unselected Korean patients with gastric cancer (2/107, 1.87%) 7. They were diagnosed with late-onset gastric cancer at 66 and 75 years, respectively. The V832M variant has been identified in a Japanese HDGC family, and the three-affected members of the family had the same mutation 8. The mutation has been shown to disrupt the ability of E-cadherin to mediate cell adhesion and to suppress cell invasion in previous functional studies 9-11.

Based on these findings, we hypothesized that the *CDH1* V832M mutation would be prevalent in Korean sporadic gastric cancer patients. The objective of this study was to assess the frequency of *CDH1* V832M mutation carriers in unselected Korean gastric cancer patients with a high incidence of sporadic gastric cancer. We investigated whether gastric cancer patients with a *CDH1* pathogenic missense mutation, V832M, actually showed characteristics of HDGC; familial gastric cancer, early-onset gastric cancer, and diffuse histology.

## Materials and Methods

### Patient and samples

Samples from 305 Korean patients who were histologically diagnosed with gastric cancer were provided by the Korea Institute of Radiological and Medical Sciences (KIRAMS) Radiation Biobank (KRB), Yonsei University Wonju Medical Center Biobank, and Samkwang Medical Laboratory Biobank. This study protocol was approved by the institutional review board. The samples were unselected for age at diagnosis and family history of gastric cancer. Clinical information including patient demographics, tumor histopathology, and family history of cancer was provided from the sample source institutions. The tumor histology was classified according to Lauren's criteria 12 and the 2010 WHO classification system 13. The stage of tumor was assigned according to the 7th edition of the American Joint Committee on Cancer TNM classification system 14. Information about family history of cancer and other cancer histories in a patient was obtained from 198 patients. A family history of gastric cancer is defined as a gastric cancer patient having one or more first- or second-degree relatives with gastric cancer. Early- onset gastric cancer is defined as a gastric cancer patient diagnosed at or before the age of 45 years; late-onset gastric cancer was defined as gastric cancer patients diagnosed over the age of 45 years.

### CDH1 V832M genotyping

Genomic DNA was extracted from buffy-coats or normal tissues using the QIAamp DNA Mini kit (Qiagen, Hilden, Germany). The primer sequences targeting codon 814 to 919 of *CDH1* exon 16 were designed using Primer3 software. The PCR was performed on 100 ng of genomic DNA, and sequencing was done with the Big Dye Terminator v3.1 Cycle Sequencing Ready Reaction Kit (Applied Biosystems, Foster City, CA, USA) on the ABI 3500 Genetic Analyzer (Applied Biosystems). The results were compared with the reference sequence using Sequencher 5.1 software (Gene Codes Corp., Ann Arbor, MI, USA).

### Statistical analysis

Statistical analysis was performed using SPSS Statistics version 24.0.0 (IBM Corp., Armonk, NY, USA). For comparison between mutation carriers and non-carriers, we used Fisher's exact test for categorycal variables and the Mann-Whitney U test for continuous variables. All *p-*values were two-tailed, and values less than 0.05 were considered statistically significant.

## Results

Clinicopathological data for 305 unselected Korean gastric cancer patients are summarized in Table 1. The study included 194 males and 111 females, with a median age of 64 years (range, 31-91 years). This series included 227 intestinal types (74.4%), 56 diffuse types (18.4%), and 22 mixed types (7.2%) in accordance with Lauren's classification. According to the AJCC/UICC criteria, there were 151 cases in stage I (49.5%), 55 cases in stage II (18.0%), 83 cases in stage III (27.2%), and 16 cases (5.2%) in stage IV. Among 198 patients with information about a family history and history of cancer, 37 patients (18.7%) had family history of cancer in first- or second- degree relatives and 13 patients had a past history of cancer other than gastric cancer (6.6%). Early-onset cases (age of cancer diagnosis ≤45 years) represented 9.5% of total participants.

In the present study, we found seven carriers of the *CDH1* V832M mutation (carrier frequency = 0.0230, 7/305) (Table 2). The carrier frequencies peaked at 60-69 years (0.0361), followed by 50-59 years (0.0274) (Table 3). There was one case (P186) of early-onset (≤45 years) cancer with the V832M mutation. The patient was diagnosed with intestinal- type gastric adenocarcinoma at 41 years and had a family history of gastric cancer in his father. There were two cases (P41 and P253) with diffuse-type cancer histology and V832M mutation, and neither case had a known family history of gastric cancer.

We compared the known phenotypic characteristics of HDGC by V832M carrier status (Table 4). The median age of cancer diagnosis was not different among V832M carriers *vs*. non-carriers (62 *vs.* 64 years, respectively, *P* value = 0.477, Mann-Whitney U test). The diffuse or mixed type histology based on Lauren's classification was not significantly associated with the V832M carrier status (*p*-value >0.999, Fisher's exact test). Moreover, a family history of gastric cancer, age of cancer diagnosis, and other cancer histories in a patient was also not significantly associated with the V832M carrier status.

## Discussion

The *CDH1* gene encodes an epithelial cadherin protein, E-cadherin. E-cadherin plays roles in regulating cell-cell adhesion, mobility and proliferation of epithelial cells 15. Loss of function of E-cadherin impairs epithelial cell adhesiveness and contributes to cancer progression and metastasis 16. The germline pathogenic truncating and missense mutations are distributed throughout the entire *CDH1* gene, and the V832M mutation is located in exon 16, the cytoplasmic domain of the protein 17. The V832M mutation was first identified in a Japanese HDGC family, and was reported again in a patient with early-onset lobular breast cancer 8,18. This missense mutation has been shown to affect the role of E-cadherin in mediating cell adhesion and suppressing cell invasion 9-11.

Current clinical guidelines regarding HDGC recommend prophylactic total gastrectomy for *CDH1* pathogenic mutation carriers due to the observation of early gastric cancer in prophylactic gastrectomy samples from *CDH1* mutation carriers 2,3. *CDH1* germline mutations have been described in about 40% of HDGC families and about 2.3% of sporadic early-onset gastric cancer patients 1. Recently, Lo *et al* 17 reported that the location and type of germline *CDH1* variants are related to the age of cancer onset and risk of concomitant cancer in the HDGC families. However, data on the frequency and clinical characteristics of germline *CDH1* mutations in unselected gastric cancer patients are scarce. Due to limited data, policies for genetic counseling and management in germline* CDH1* mutation carriers in sporadic gastric cancer have not been established. As panel testing increases in popularity, *CDH1* variants might be identified in patients who do not meet HDGC criteria 6,7,19. Huynh *et al.* reported two families with *CDH1* mutations that did not meet any of the International Gastric Cancer Linkage Consortium (IGCLC) criteria 6. The two families with a nonsense mutation and a splicing mutation in *CDH1*, respectively, did not have a family history of diffuse gastric cancer 6. These findings suggest that a different approach is needed when a *CDH1* mutation is found in a sporadic or asymptomatic setting.

Frequencies of *CDH1* germline mutations in gastric cancer have been known to vary according to the incidence of gastric cancer 3,20. Corso *et al.* reported that areas with a high risk of gastric cancer show a higher frequency of *CDH1* missense mutations than non-missense mutations 20. Current clinical guidelines are based primarily on data from areas with a low-incidence of sporadic gastric cancer and truncating mutations 3. Based on our prior observation 7, we searched the V832M mutation in unselected Korean gastric cancer patients, and the mutation was found regardless of the age of cancer diagnosis, family history of gastric cancer, or Lauren's histology type.

The *CDH1* V832M allele is common in Koreans and Japanese, with reported population frequencies of 0.016 (30/1909) and 0.013 (1/76), respectively, in the gnomad database (http://gnomad.broadinstitute. org). The expected number of carriers was not different from the numbers observed in this study (5.10 vs. 7, *P*=0.360, χ^2^ test). The reason why the V832M mutation is so frequent in the population database is that the variant might have low-penetrance and late-onset characteristics, which encompass many carriers in the control population. To estimate the cancer risk associated with the V832M mutation, further studies using cancer-free age-matched controls are needed.

The limitation of this study is that we could not get information about histology type in cases with a family history of gastric cancer. Since our study was conducted retrospectively and the series had not been fully evaluated for HDGC clinical criteria, HDGC was not completely excluded in our series. Future studies are needed with more patients with a detailed family history.

To date, studies have not evaluated known *CDH1* missense mutations in a large number of unselected gastric cancer patients to determine the statistical association with clinical features. We firstly proved that a known missense mutation, V832M, is not associated with clinical features previously known as characteristics of patients with *CDH1* mutation carriers. Especially our study is directly relevant to East-Asians who are common with the V832M allele. It also suggests that the same approach is needed for missense mutations common to other races.

Our study shows that a germline *CDH1* V832M mutation is commonly found in sporadic, late onset, or intestinal type gastric cancer, as well as familial, early onset, or diffuse type gastric cancer. These findings suggest that the presence of the V832M mutation is not an indicator of HDGC diagnosis or early-onset of gastric cancer in a patient. Therefore, if the V832M mutation is found in patients who do not meet the HDGC clinical criteria, the risk prediction and management, including preventive surgery, need to be differentiated from that of HDGC patients. It is important to supplement the management protocol based on more data on *CDH1* mutation penetrance in sporadic gastric cancer cases among different geographic regions.

## Figures and Tables

**Table 1:** Clinicopathological characteristics of patients with gastric cancer

Variables		All patients (*N*=305)^a^
**Age (years)**		64 (53.5, 74)
**Sex**		
	Female	111 (36.4%)
	Male	194 (63.6%)
**Lauren's classification**		
	Diffuse	56 (18.4%)
	Intestinal	227 (74.4%)
	Mixed	22 (7.2%)
**WHO classification**		
	Mucinous	5 (1.6%)
	Tubular	239 (78.4%)
	Poorly cohesive	57 (18.7%)
	Mixed (tubular and poorly cohesive)	2 (0.7%)
	Mixed (tubular and papillary)	1 (0.3%)
	Adenosquamous	1 (0.3%)
**TNM classification**		
	Stages IA/IB	114 (37.4%)/37 (12.1%)
	Stages IIA/IIB	30 (9.8%)/25 (8.2%)
	Stages IIIA/IIIB/IIIC	34 (11.1%)/22 (7.2%)/27(8.9%)
	Stages IV	16 (5.2%)
**Family history of gastric cancer**		
	2 family members	5 (1.6%)
	1 family member	32 (10.5%)
	No family history	161 (52.8%)
	Unknown	107 (35.1%)
**Age of cancer diagnosis**		
	Early-onset (≤45 years)	29 (9.5%)
	Late-onset (>45 years)	276 (90.5%)
**Other cancer histories in a patient**		
	Breast cancer	2 (0.7%)
	Colon cancer	3 (1.0%)
	Other cancer^b^	8 (2.6%)
	None	185 (60.7%)
	Unknown	107 (35.1%)

^a^Results are expressed as median (interquartile ranges) or number (%).^b^Two patients with urinary tract cancer, a patient with essential thrombocythemia, a patient with pharyngeal cancer, a patient with lung cancer, a patient with papillary thyroid cancer, a patient with ovary cancer, and a patient with hepatocellular carcinoma and cervix cancer.

**Table 2:** Korean gastric cancer patients with CDH1 V832M germline mutation identified in this study

ID	Sex	Age at diagnosis	Lauren's classification	WHO classification	TNM classification	EBV infection^a^	MSI state^b^	*H. pylori* infection^c^	Family history of cancer (affected family member)	Other cancer histories in a patient (age at diagnosis)
**P41**	F	50	Diffuse	Poorly cohesive	Stage IA	Negative	MSS	Negative	None	Papillary thyroid cancer (49)
**P93**	F	58	Intestinal	Tubular	Stage IA	Negative	MSS	Not done	Colon cancer (a brother)	None
**P119**	M	62	Intestinal	Tubular	Stage IIIA	Positive	MSS	Not done	None	None
**P143**	F	66	Intestinal	Tubular	Stage IIA	Negative	MSI-H	Not done	Lymphoma (a son)	None
**P154**	M	66	Intestinal	Tubular	Stage IIB	Negative	MSS	Positive	Unknown	Unknown
**P186**	M	41	Intestinal	Tubular	Stage IA	Negative	MSS	Negative	Gastric cancer (a father)	None
**P253**	M	75	Diffuse	Tubular	Stage IIB	Negative	MSI-H	Negative	Unknown	Unknown

EBV, Epstein-Barr virus; MSI, microsatellite instability; *H. pylori*, *Helicobacter pylori*; MSS, microsatellite stability; MSI-H, microsatellite instability high.^ a^EBV infection was detected using in-situ hybridization or real-time PCR.^ b^Microsatellite instability status was determined by the mononucleotide repeat markers NR-21, BAT-26, BAT-25, NR-24, and NR-27. MSI-H was defined as a tumor with two or more of the five markers of instability, and MSS was defined as the absence of any marker.^ c^*H. Pylori* infection was detected using Giemsa stain or PCR and sequencing.

**Table 3:** Frequencies of CDH1 V832M mutation as related to the age at gastric cancer diagnosis

Age at diagnosis	No. of patients	No. of carriers (carrier frequency)
<40	6	0
40-49	43	1 (0.0233)
50-59	73	2 (0.0274)
60-69	83	3 (0.0361)
70-79	78	1 (0.0128)
≥80	22	0
Total	305	7 (0.0230)

**Table 4:** Association of the *CDH1* V832M germline mutation and phenotypes in gastric cancer patients

Variables		V832M heterozygotes (*N*=7)^a^	V832M non-carrier (*N*=298)^a^	*P* value^b^
**Age (years)**		62 (54, 66)	64 (54,74)	0.477
**Sex**				0.708
	Female	3 (42.9%)	108 (36.2%)	
	Male	4 (57.1%)	190 (63.8%)	
**Lauren's classification**				>0.999
	Diffuse/mixed type	2 (28.6%)	76 (25.5%)	
	Intestinal type	5 (71.4%)	222 (74.5%)	
**Family history of gastric cancer^c^**				>0.999
	Presence (one or more family members)	1 (20.0%)	36 (18.7%)	
	Absence	4 (80.0%)	157 (81.3%)	
**Age of cancer diagnosis**				0.507
	Early-onset (≤45 years)	1 (14.3%)	28 (9.4%)	
	Late-onset (>45 years)	6 (85.7%)	270 (90.6%)	
**Other cancer histories in a patient^c^**				0.290
	Presence	1 (20.0%)	12 (6.2%)	
	Absence	4 (80.0%)	181 (93.8%)	

^a^Results are expressed as median (interquartile ranges) or number (%). ^b^The* P* values were calculated by Mann-Whitney U test for continuous variables and Fisher's exact test for categorical variables.^ c^Family history of gastric cancer and other cancer history in a patient were assessed in 198 patients.
